# Actively Controllable Terahertz Metal–Graphene Metamaterial Based on Electromagnetically Induced Transparency Effect

**DOI:** 10.3390/nano12203672

**Published:** 2022-10-19

**Authors:** Liang Gao, Chao Feng, Yongfu Li, Xiaohan Chen, Qingpu Wang, Xian Zhao

**Affiliations:** 1 Center for Optics Research and Engineering, Key Laboratory of Laser & Infrared System, Ministry of Education, Shandong University, Qingdao 266237, China; 2 Shandong Provincial Key Laboratory of Laser Technology and Application, School of Information Science and Engineering, Shandong University, Qingdao 266237, China

**Keywords:** terahertz, graphene, metamaterials, electromagnetically induced transparency

## Abstract

A metal–graphene metamaterial device exhibiting a tunable, electromagnetically induced transparency (EIT) spectral response at terahertz frequencies is investigated. The metamaterial structure is composed of a strip and a ring resonator, which serve as the bright and dark mode to induce the EIT effect. By employing the variable conductivity of graphene to dampen the dark resonator, the response frequency of the device shifts dynamically over 100 GHz, which satisfies the convenient post-fabrication tunability requirement. The slow-light behavior of the proposed device is also analyzed with the maximum group delay of 1.2 ps. The sensing performance is lastly studied and the sensitivity can reach up to 100 GHz/(RIU), with a figure of merit (FOM) value exceeding 4 ${\mathrm{RIU}}^{-1}$. Therefore, the graphene-based metamaterial provides a new miniaturized platform to facilitate the development of terahertz modulators, sensors, and slow-light applications.

## 1. Introduction

Terahertz (THz) radiation has many unique applications in sensing, imaging, spectroscopic analysis, and communications [1,2]. The development of terahertz sources and detectors, such as quantum-cascade lasers (QCLs) [3,4] and quantum-well photodetectors [5], as well as broadband time-domain spectroscopy (TDS) systems [6], has made many terahertz applications available. Nevertheless, in order to fully exploit THz applications, external modulators that can actively manipulate THz properties and simultaneously work in conjunction well with standardized sources and detectors are required. Metamaterials as a new class of artificial structure constructed by the sub-wavelength building elements have been utilized to engineer artificial media with properties unavailable in nature [7,8,9,10,11,12,13,14,15,16,17]. Furthermore, metamaterials are promising candidates to satisfy the external THz modulator’s requirements thanks to the collective resonance and strong light–matter interaction in the internal structures [7,8,9,10,11,12]. However, the response range of most predesigned metamaterials is very narrow when excited by a varying incident field, which limits many practical applications [18]. Therefore, various metamaterial structures integrated with conductivity variable materials are investigated to realize the dynamical control of the device response [19,20,21,22,23,24]. Among those conductivity changeable materials, graphene is a promising candidate for modulation as it possesses a wide conductivity change range when responding to external stimuli and is easily integrated into the metamaterial fabrication process [25,26].

In recent years, actively controllable graphene-based metamaterials have been widely studied at terahertz frequencies [19,20,21,22,27,28,29]. Nonetheless, there are only a few reports about resonant frequency tunable devices based on graphene resonant features. Ref. [29] demonstrated a THz metal–graphene frequency modulator showing dynamically controllable single, double, and multiple transmission windows. A multi-layer metamaterial incorporated with graphene cross structures was also developed to realize frequency and amplitude modulation [30]. Both of the above methods investigated the shift of the resonance frequency by adjusting the Fermi energy of graphene. However, the frequency tuning range is relatively narrow, only several tens of GHz, which constrains the application scopes of THz radiation.

In order to enlarge the frequency tuning range, we designed a metamaterial structure consisting of a strip and a ring resonator integrated with graphene, which demonstrated a frequency tuning range over 100 GHz based on the electromagnetically induced transparency (EIT) effect. The main advantage of this method is that it exploits the tunable loss of graphene, instead of high carrier mobility, to realize frequency tuning. When THz wave incidents on the device surface, the strip resonator couples strongly with the incident field and supports a bright mode, whereas the ring resonator is indirectly activated by the bright mode through near-field coupling and supports a dark mode. The bright–dark mode coupling induces the electromagnetically induced transparency effect [31,32]. The two resonators exhibit a strong THz response, while graphene is used to variably dampen the strength of THz response. With the loss in the graphene-dampened ring resonator increasing, the resonance condition is changed from a strongly coupled system to a single resonator system, which leads to over 100 GHz frequency tuning. Metamaterial devices based on the EIT effect have attracted much attention due to the applications in optical data storage [33], nonlinear optical enhancement [34], and ultrasensitive biosensing [35,36]. The proposed metal–graphene device can also modify the group delay due to its inherent reconfigurable dispersion, which has important slow-light applications for optoelectronic devices. The maximum group delay for this device is calculated to be 1.2 ps at 1.64 THz. In addition, the device has potential applications in tunable sensors and selective filters. Placing an analyte above the metamaterial and changing its refractive index from 1 to 2.4, the resonance frequency shifts from 1.56 THz to 1.4 THz. The corresponding sensitivity and best FOM value are 100 GHz/(RIU) and 4 ${\mathrm{RIU}}^{-1}$, respectively, where RIU is refractive index unit. Integrating the proposed metal–graphene device with standard sources and detectors would consequently promote THz applications such as spectroscopy, light storage, and sensing fields. Particularly, the metal–graphene based device can also be potentially used in biomedicine, sensing, and diagnostics, due to its high sensitivity mechanism and label-free and rapid detection, together with the graphene’s large surface-to-volume ratio and biocompatibility. Furthermore, the device can be utilized as a continuously tunable fast band pass filter or as a band-stop filter in the THz range. A frequency modulator for THz communications protocols such as frequency-shift keying is also a potential application.

## 2. Device Design and Frequency Tuning

**Figure 1:**
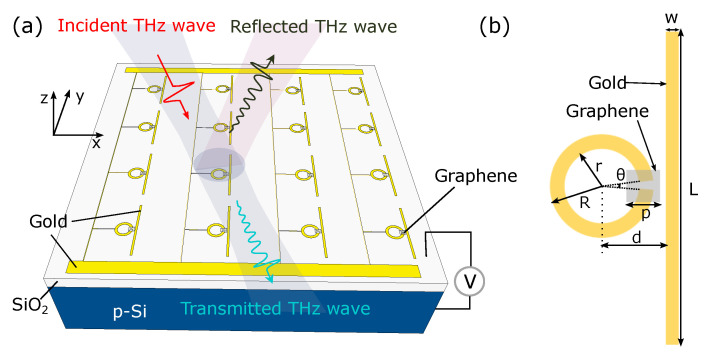
(**a**) The schematic diagram of overall device architecture with electrostatic back gating. The backside of the device coated with gold bond pads is connected to the ground. The DC conductivity of graphene is a function of the back-gated voltage, V. (**b**) Representative design of a unit cell. The geometrical parameters are L = 29 μm, W = 1.5 μm, R = 5.5 μm, r = 4 μm, p = 3 μm, d = 7 μm, and θ = 10${}^{\circ }$, respectively.

Figure 1a shows a representative array of the proposed design. The 300 nm insulating layer (${\mathrm{SiO}}_{2}$) is grown on the p-doped silicon substrate to realize the electrostatic back gate. The metal–graphene metamaterial can be fabricated on the top of the ${\mathrm{SiO}}_{2}$ layer. The backside of the whole structure is coated with gold bonding pads in order to connect to the ground. The finite element method is employed in order to calculate the transmission properties and electric field distributions of the proposed structure. The ${\mathrm{SiO}}_{2}$ layer is assumed to be a perfect dielectric, and the permittivity is set to 9.1. The silicon substrate has a standard permittivity of 11.56. The electric field placed on the top is configured to transmit incident radiation with a given angular frequency, ω, and a nominal power of 1 W, with the electric field polarized along the strip (y direction). The measured electric field amplitude in the y direction at the bottom is used to determine the transmitted power through the sample. The perfectly matched layers (PML) absorbing condition is set in the propagation directions (z direction) of the normal incidence plane wave, and the periodic boundary condition is used in the x and y directions to model the periodic array. In order to ensure the simulation accuracy, the mesh shape is chosen as triangular and the maximum mesh element size of each domain is set to roughly no larger than tenth of the material wavelength.

The graphene conductivity consisting of the interband and intraband contributions is derived from Kubo’s formula: $\sigma \left(\omega \right)=\sigma_{inter}\left(\omega \right)+\sigma_{intra}\left(\omega \right)$. In the terahertz regime, the graphene conductivity is mainly determined by intraband transitions [37,38], which can be described by a Drude-like form,

(1) $$\sigma_{intra}=\frac{je^{2}\mu_{c}}{\pi \hbar^{2}\left(\omega +j\tau^{-1}\right)}=\frac{\sigma_{0}}{1-j\omega \tau }$$

where *j* is the imaginary unit, *e* is electron charge, *ħ* is the reduced Planck’s constant, and τ is the Drude scattering time. $\mu_{c}$ is the chemical potential that is related to the Fermi energy ($E_{F}$) and Fermi velocity ($v_{F}$) of graphene. By tuning the external applied bias voltage, graphene’s chemical potential can be changed significantly and thus the transmission properties of graphene-based devices can be controlled. The DC conductivity ($\sigma_{0}$), which is related to chemical potential, is also a function of back-gate voltage based on Equation (1). For simplicity, the DC graphene conductivity is used for the following simulation results when describing various graphene conductivities with average Drude scattering time values of 50 fs [39]. For gold, the DC conductivity and the average Drude scattering time values are given as $2.7\times 10^{2}$ mS and 200 fs, respectively [40].

**Figure 2:**
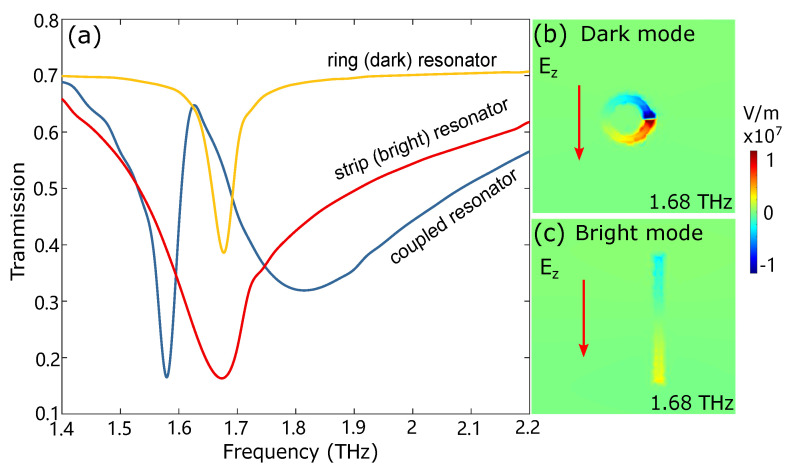
(**a**) Transmission spectra of the strip resonator (red), ring resonator (yellow), and coupled strip/ring resonator (blue) without graphene. The transmission dips of ring and strip resonator are both at 1.68 THz. The EIT induced transparency window due to bright–dark-mode coupling is at 1.62 THz. (**b**,**c**) The electric field $E_{z}$ distribution of the dark resonator and bright resonator at the transmission dips when they are excited by the incident field separately. The arrows represent the current directions in the resonators.

Figure 1b demonstrates a unit cell of the proposed design. The length and width of the strip resonator are 29 μm and 1.5 μm, respectively. The radiuses of the inner and outer ring are 5.5 μm and 4 μm, respectively. The opening angle of the slit is 10${}^{\circ }$. The distance from the strip to the center of the ring resonator is 7 μm. The side length of the square graphene is 3 μm. The strip resonator on the right acts as a bright resonator that can strongly couple with the incident THz radiation. In order to realize high coupling efficiency, the polarization of the incident electric field is set to parallel to the strip resonator (y direction) since the excitation efficiency of the resonance mode is strongly dependent on the electric field polarization. The excited resonance mode of the bright resonator demonstrates a broad transmission dip due to the strong radiative losses as shown in Figure 2a. The ring resonator on the left, with a small capacitive gap, is a dark resonator that is mainly excited by the near-field capacitive coupling with the bright resonator, and it shows a narrow transmission dip due to the weak coupling to the incident field. The resonace frequencies of the bright and dark modes are both at 1.68 THz. The electric field $E_{z}$ distribution, collected 20 nm above the metal–graphene metamaterial, of the dark resonator and bright resonator at 1.68 THz is shown in Figure 2b,c, in which it is excited by the incident field separately. The polarity of charges on the two resonators is in the same direction and shows conventional localized surface plasmon resonances.

**Figure 3:**
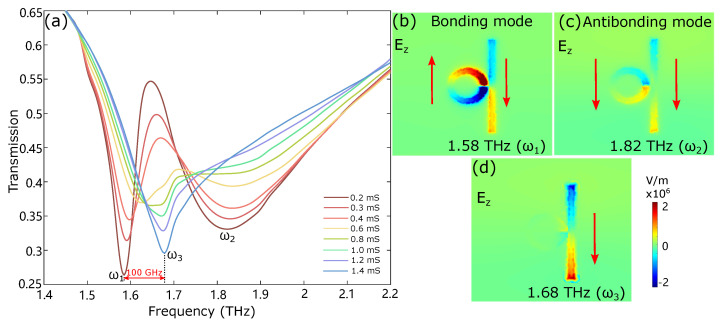
(**a**) Transmission spectra of the metal–graphene device for different DC graphene conductivities. With the increase in the graphene conductivity from 0.2 mS to 1.4 mS, the resonance frequency is tuned from 1.58 THz ($\omega_{1}$) to 1.68 THz ($\omega_{3}$), with a frequency tuning range of 100 GHz. (**b**,**c**) The electric field $E_{z}$ distribution of the coupled device at two transmission dips at frequency of 1.58 THz ($\omega_{1}$) and 1.82 THz ($\omega_{2}$). The different induced current directions in the two resonators illustrate that the low-energy mode is bonding mode while the high-energy mode is the antibonding mode of the coupled structure. (**d**) The electric field $E_{z}$ distribution of the coupled device is 1.68 THz when the conductivity of graphene is set at 1.4 mS. Only the bright resonator shows strong localized surface plasmon resonance, while the resonance of the dark resonator is much weaker.

The Q factors ($Q=f_{0}/FWHM$, $f_{0}$ is the center frequency, and FWHM is the full width at half maximum) of the strip and ring resonator are calculated to be 6.87 and 34.69, respectively. The similar resonance frequency and large contrast of the Q factors between these two coupling modes lead to two splitting transmission dips and a sharp transmission peak due to the EIT effect, as shown in Figure 2a, when there is no integration with graphene. Similar transmission spectra are also achieved when the DC conductivity of the graphene is small, as shown in Figure 3a. The low-energy mode at 1.58 THz ($\omega_{1}$) possesses the opposite induced current directions in two resonators (Figure 3b), analog to the bonding mode in a hybridized moelcular system. The high-energy mode at 1.82 THz ($\omega_{2}$) exhibits the induced currents in a similar direction (Figure 3c), which can be directly associated with the antibonding mode in a hybridized molecular system. The sharp transmission peak at 1.62 THz within two hybridized modes is due to the direct destructive interference between the bright and the dark modes. Figure 3b,c illustrate the electric field $E_{z}$ distribution of the bonding mode and antibonding mode. The electric field is concentrated in the bright and dark resonators, and the current induced in the bonding mode condition is about 2 times higher than the antibonding mode condition for the same incident power.

Figure 3a describes the transmission spectra of the coupled resonator device as a function of frequency with different DC graphene conductivities (The corresponding reflection and phase properties are shown in Figures S1 and S2 of the Supplemental Materials). With the increase in graphene conductivites, the strength of antibonding resonace reduces quickly, whereas the bonding resonance is continuously blueshifted until the single resonator resonance takes over. The dampening of the dark resonator is increased as the conductivity of the graphene is changed from 0.2 mS to 1.4 mS via electrostatic back gating, switching the metamaterial from a coupled resonator system to a single resonator system. The bonding resonance frequency is correspondingly tuned from 1.58 THz to 1.68 THz. Figure 3d shows the electric field $E_{z}$ distribution of the coupled resonator at 1.68 THz when the DC graphene conductivity is set at 1.4 mS. There is a strong localized surface plasmon resonance in the bright resonator that is similar to the isolated bright resonator case in Figure 2c. The electric field in the dark resonator is now an order of magnitude smaller, confirming that the coupling system has been transformed into a single resonator system. As a consequence, a continuous frequency tuning range of 100 GHz is achieved, with the graphene conductivity changed from 0.2 mS to 1.4 mS. An equivalent circuit model [41,42,43] is also utilized to investigate the resonance properties of the metal-graphene device, see Figures S3 and S4 and Table S1 in the Supplemental Materials.

## 3. Group Delay

In the EIT-like effect induced-transparency window, the phase experiences steep variation, leading to strong dispersion and tremendous group delay. This indicates that when a light pulse with a center frequency situated in the transparency window travels through the metamaterial, the light group velocity will be considerably slowed down. The ability to actively control slow light attracts much interest due to its implications for fundamental scientific research as well as optical technique implementation. The group delay ($t_{g}$) and group index ($n_{g}$) are the two key parameters to qualify this phenomenon in these devices.

**Figure 4:**
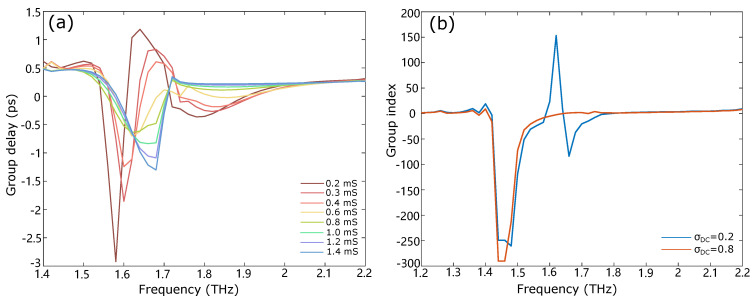
(**a**) Group delay of the metal–graphene device at different DC graphene conductivities. The maximum positive group delay is 1.2 ps at 1.64 THz when $\sigma_{DC}$ = 0.2 mS. The group delay is reduced with the increase in the graphene conductivity. (**b**) The group index plot at two different graphene conductivities. When $\sigma_{DC}$ = 0.2 mS, the value of $n_{g}$ is larger than 150 within the EIT transparency window, while $n_{g}$ is near 0 when $\sigma_{DC}$ = 0.8 mS.

The group delay, $t_{g}=\frac{d\Phi }{2\pi df}$, is the time delay of a THz wave packet through the sample in comparison to air, where Φ is the phase shift introduced by the EIT effect. Figure 4a shows the group delays of the proposed structure with different graphene conductivity. The region of interest for slow light is for the frequencies when the group delay is positive. The maximum positive group delay is achieved with graphene conductivity $\sigma_{DC}=0.2$ mS. There occurs a strong dispersion, leading to a large group delay up to 1.2 ps at 1.64 THz, which means trapping photons for a long time inside the structure. The large positive group delay of the proposed metamaterials will be potentially used in the field of routing optical information and enhancing light–matter interactions. The group delays of the EIT transparency peaks are suppressed with the increase in the graphene conductivity due to the greater dissipative power density. When $\sigma_{DC}$ is above 0.6 mS, the group delay is negative or around 0 for all frequencies and cannot induce the slow-light effect in the proposed structure.

The group index of the device can be retrieved from the transmission and reflection coefficients following the methods in Refs. [44,45]. Figure 4b shows the group index values for two different graphene conductivities. When $\sigma_{DC}$ = 0.2 mS, the value of $n_{g}$ exceeds 150 within the EIT transparency window, whereas the group index is near 0 for $\sigma_{DC}$ = 0.8 mS, which means the large graphene conductivity is not suitable for realizing the slow-light effect. The group delay and group index results clearly demonstrate that the proposed design has the potential to be used in slow-light applications.

## 4. Sensing

The EIT-like response of the proposed metamaterial is sensitive to the change of the refractive index of the ambient medium due to the intensive localization of electromagnetic energy inside the coupled structure. Therefore, the metamaterial-based sensors have the ability to broke the resolution limit of traditional THz time-domain spectroscopy. When the sample is placed on top of the metal–graphene structure, its resonance frequency changes correspondingly. The shift of resonance frequency is caused by the concentration, permittivity, or thickness changes of the surrounding medium. To detect the change of sample properties more accurately, the realization of the sensor with high performance is especially critical. The sensing performance can be quantified using the spectral sensitivity (S) and a figure of merit (FOM) as the quality factors [35]. The spectral sensitivity is defined as S=Δf/Δn, where Δn is the change of refractive index of the sample and Δf is the resonance frequency shift. The figure of merit, FOM=S/FWHM, can simultaneously characterize the sensitivity and resolution of the sensor. A higher sensitivity and smaller FWHM means better sensing performance.

**Figure 5:**
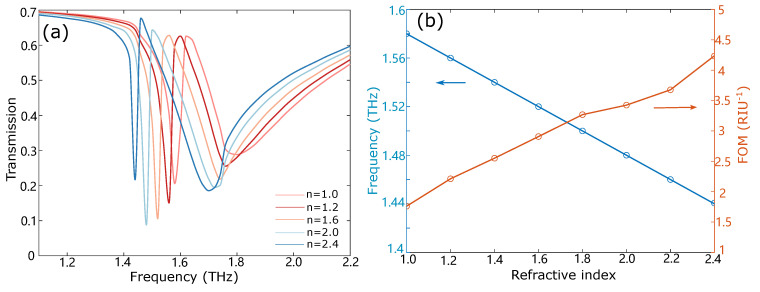
(**a**) Transmission spectra of the metal–graphene metamaterial with different refractive indexes of analyte. The resonance frequency shifts from 1.58 THz to 1.44 Thz with the refractive index of analyte changes from 1 to 2.4. (**b**) Resonance frequency and FOM as a function of the refractive index of analyte. The resonance frequency linearly depends on the refractive index. The maximum FOM value is 4.24 ${\mathrm{RIU}}^{-1}$ when the refractive index of analyte is 2.4.

As shown in Figure 5a, when the refractive index of the analyte changes from 1 to 2.4, the resonance frequency undergoes a significant red shift from 1.58 THz to 1.44 THz. The frequency change of the proposed structure linearly depends on the refractive index of the covering analyte in a wide detection range, which can be readily employed as an excellent zero-order refractive-index-based sensor. The sensitivity is calculated to be 100 GHz/RIU. In addition, the corresponding FOM values are also calculated and shown in Figure 5b. With the increase in the analyte refractive index, the localized field of the metamaterial is more strongly confined inside the structure, leading to small FWHM and large FOM values. Thus, the proposed structure demonstrates high sensitivity and is particularly attractive for thin-film and biomedical sensing applications [46].

## 5. Conclusions

In conclusion, a dynamically controllable terahertz metamaterial consisting of a strip and a ring resonator with graphene integration is investigated. The strip resonator is strongly excited by the incident field and serves as bright mode, while the ring resonator is excited through near-filed capacitive coupling with the bright resonator and serves as dark mode. The bright–dark mode coupling induces the EIT effect. The resonance frequency of the device is tuned over 100 GHz by adjusting the graphene conductivity to dampen the dark resonator. The slow-light behavior of the device is also studied with the maximum group delay of 1.2 ps. Furthermore, the device can be utilized as an effective refractive index sensor with a sensitivity of 100 GHz/RIU and a FOM value above 4 ${\mathrm{RIU}}^{-1}$. Therefore, a tunable photonic platform based on a metal–graphene metamaterial is developed, demonstrating the potential to advance the development of, for example, THz frequency modulators, tunable optical retarders, and biochemical sensors. In the future, the device could be fabricated following the design parameters. The conductivity of the graphene sheet as a function of backgate voltage should be firstly tested. The frequency-dependent transmission properties of the device should be measured using a TDS method and compared with the simulation data in order to optimize the design parameters. In addition, the frequency tuning range of the proposed device is limited to ∼100 GHz, which limits its applications requiring a large frequency tuning range. In the next study, different coupling schemes and various 2D materials should be developed in order to enlarge the frequency tuning range further.

## Data Availability

The data presented in this paper are available on reasonable request from the corresponding author.

## Supplementary Materials

The following supporting information can be downloaded at: https://www.mdpi.com/article/10.3390/nano12203672/s1, Figure S1: Reflection spectra of the metal-graphene device for different DC graphene conductivities; Figure S2: Phase properties of the metal-graphene device for different DC graphene conductivities; Figure S3: Equivalent circuit model of the metal-graphene coupled devices; Figure S4: The calculate transmission spectra based on the equivalent circuit model for different DC graphene conductivities; Table S1: The parameter values of the equivalent circuit.

## References

[1] Tonouchi M. (2007). Cutting-edge terahertz technology. Nat. Photonics.

[2] Dhillon S., Vitiello M., Linfield E., Davies A., Hoffmann M.C., Booske J., Paoloni C., Gensch M., Weightman P., Williams G. (2017). The 2017 terahertz science and technology roadmap. J. Phys. D Appl. Phys..

[3] Williams B.S. (2007). Terahertz quantum-cascade lasers. Nat. Photonics.

[4] Vitiello M.S., Scalari G., Williams B., Natale P.D. (2015). Quantum cascade lasers: 20 years of challenges. Opt. Express.

[5] Guo X.G., Cao J.C., Zhang R., Tan Z.Y., Liu H.C. (2013). Recent Progress in Terahertz Quantum-Well Photodetectors. IEEE J. Sel. Top. Quantum Electron..

[6] Ferguson B., Zhang X.C. (2006). Materials for terahertz science and technology. Nat. Mater..

[7] Pendry J.B., Holden A.J., Stewart W.J., Youngs I.I. (1996). Extremely Low Frequency Plasmons in Metallic Mesostructures. Phys. Rev. Lett..

[8] Smith D.R., Pendry J.B., Wiltshire M.C. (2004). Metamaterials and Negative Refractive Index. Science.

[9] Gollub J.N., Smith D.R., Vier D.C., Perram T., Mock J.J. (2005). Experimental characterization of magnetic surface plasmons on metamaterials with negative permeability. Phys. Rev. B.

[10] Schurig D., Mock J.J., Justice B.J., Cummer S.A., Pendry J.B., Starr A.F., Smith D.R. (2006). Metamaterial Electromagnetic Cloak at Microwave Frequencies. Science.

[11] Danila O. (2021). Polyvinylidene Fluoride-Based Metasurface for High-Quality Active Switching and Spectrum Shaping in the Terahertz G-Band. Polymers.

[12] Danila O., Manaila-Maximean D., Arar A., Loiko V.A. (2021). Non-Layered Gold-Silicon and All-Silicon Frequency-Selective Metasurfaces for Potential Mid-Infrared Sensing Applications. Sensors.

[13] Estakhri N.M., Edwards B., Engheta N. (2019). Inverse-designed metastructures that solve equations. Science.

[14] Spada L.L., Vegni L. (2017). Near-zero-index wires. Opt. Express.

[15] Greybush N.J., Pacheco-Peña V., Engheta N., Murray C.B., Kagan C.R. (2019). Plasmonic Optical and Chiroptical Response of Self-Assembled Au Nanorod Equilateral Trimers. ACS Nano.

[16] Lalegani Z., Ebrahimi S.A.S., Hamawandi B., Spada L.L., Batili H., Toprak M.S. (2022). Targeted dielectric coating of silver nanoparticles with silica to manipulate optical properties for metasurface applications. Mater. Chem. Phys..

[17] Donnelly E., Spada L.L. (2020). Electromagnetic and thermal nanostructures: From waves to circuits. Eng. Res. Express.

[18] Padilla W.J., Taylor A.J., Highstrete C., Lee M., Averitt R.D. (2006). Dynamical electric and magnetic metamaterial response at terahertz frequencies. Phys. Rev. Lett..

[19] Miao Z., Wu Q., Xin L., He Q., Lei Z. (2015). Widely Tunable Terahertz Phase Modulation with Gate-Controlled Graphene Metasurfaces. Phys. Rev. X.

[20] Luo L., Wang K., Guo K., Shen F., Zhang X., Yin Z., Guo Z. (2017). Tunable manipulation of terahertz wavefront based on graphene metasurfaces. J. Opt..

[21] Degl’Innocenti R., Jessop D.S., Shah Y.D., Sibik J., Zeitler J.A., Kidambi P.R., Hofmann S., Beere H.E., Ritchie D.A. (2014). Low-bias terahertz amplitude modulator based on split-ring resonators and graphene. ACS Nano.

[22] Jessop D., Kindness S., Xiao L., Braeuninger-Weimer P., Lin H., Ren Y., Ren C., Hofmann S., Zeitler J., Beere H. (2016). Graphene based plasmonic terahertz amplitude modulator operating above 100 MHz. Appl. Phys. Lett..

[23] Tong S., Fyffe A., Xiao B., Shi Z. (2020). Tunable electromagnetically induced transparency based on graphene metamaterials. Opt. Express.

[24] Kindness S.J., Jessop D.S., Wei B., Wallis R., Kamboj V.S., Xiao L., Ren Y., Braeuninger-Weimer P., Aria A.I., Hofmann S. (2017). External amplitude and frequency modulation of a terahertz quantum cascade laser using metamaterial/graphene devices. Sci. Rep..

[25] Efetov D.K., Kim P. (2010). Controlling Electron-Phonon Interactions in Graphene at Ultrahigh Carrier Densities. Phys. Rev. Lett..

[26] Nikitin A.Y., Guinea F., Garcia-Vidal F.J., Martin-Moreno L. (2011). Edge and waveguide THz surface plasmon modes in graphene micro-ribbons. Phys. Rev. B.

[27] Liu P.Q., Luxmoore I.J., Mikhailov S.A., Savostianova N.A., Valmorra F., Faist J., Nash G.R. (2015). Highly tunable hybrid metamaterials employing split-ring resonators strongly coupled to graphene surface plasmons. Nat. Commun..

[28] Xiao S., Wang T., Jiang X., Yan X., Cheng L., Wang B., Xu C. (2017). Strong interaction between graphene layer and Fano resonance in terahertz metamaterials. J. Phys. D Appl. Phys..

[29] Yan X., Wang T., Xiao S., Liu T., Hou H., Cheng L., Jiang X. (2017). Dynamically controllable plasmon induced transparency based on hybrid metal–graphene metamaterials. Sci. Rep..

[30] Chen M., Xiao Z., Lv F., Cui Z., Xu Q. (2021). Dynamically tunable electromagnetically induced transparency-like effect in terahertz metamaterial based on graphene cross structures. IEEE J. Sel. Top. Quantum Electron..

[31] Chiam S.Y., Singh R., Rockstuhl C., Lederer F., Zhang W., Bettiol A.A. (2009). Analogue of Electromagnetically Induced Transparency in a Terahertz Metamaterial. Phys. Rev. B.

[32] Singh R., Rockstuhl C., Lederer F., Zhang W. (2009). Coupling between a dark and a bright eigenmode in a terahertz metamaterial. Phys. Rev..

[33] Kekatpure R.D., Barnard E.S., Cai W., Brongersma M.L. (2010). Phase-coupled plasmon-induced transparency. Phys. Rev. Lett..

[34] Wu Y., Saldana J., Zhu Y. (2005). Large enhancement of four-wave mixing by suppression of photon absorption from electromagnetically induced transparency. Phys. Rev. A.

[35] Liu N., Weiss T., Mesch M., Langguth L., Eigenthaler U., Hirscher M., Sonnichsen C., Giessen H. (2010). Planar metamaterial analogue of electromagnetically induced transparency for plasmonic sensing. Nano Lett..

[36] Liu G.D., Zhai X., Wang L.L., Lin Q., Xia S.X., Luo X., Zhao C.J. (2017). A High-Performance Refractive Index Sensor Based on Fano Resonance in Si Split-Ring Metasurface. Plasmonics.

[37] Winnerl S., Orlita M., Plochocka P., Kossacki P., Potemski M., Winzer T., Malic E., Knorr A., Sprinkle M., Berger C. (2011). Carrier relaxation in epitaxial graphene photoexcited near the Dirac point. Phys. Rev. Lett..

[38] Dawlaty J.M., Shivaraman S., Strait J., George P., Chandrashekhar M., Rana F., Spencer M.G., Veksler D., Chen Y. (2008). Measurement of the Optical Absorption Spectra of Epitaxial Graphene from Terahertz to Visible. Appl. Phys. Lett..

[39] Tan Y., Zhang Y., Bolotin K., Zhao Y., Adam S., Hwang E.H., Sarma S.D., Stormer H.L., Kim P. (2007). Measurement of scattering rate and minimum conductivity in graphene. Phys. Rev. Lett..

[40] Ordal M.A., Bell R.J., Alexander R.W., Long L.L., Querry M.R. (1985). Optical properties of fourteen metals in the infrared and far infrared: Al, Co, Cu, Au, Fe, Pb, Mo, Ni, Pd, Pt, Ag, Ti, V, and W. Appl. Opt..

[41] Meyrath T.P., Zentgraf T., Giessen H. (2007). Lorentz model for metamaterials: Optical frequency resonance circuits. Physical Review B.

[42] García-Vigueras M., Mesa F., Medina F., Rodríguez-Berral R., Gómez-Tornero J.L. (2012). Simplified Circuit Model for Arrays of Metallic Dipoles Sandwiched Between Dielectric Slabs Under Arbitrary Incidence. IEEE Trans. Antennas Propag..

[43] Amin M., Farhat M., Bagci H. (2013). A dynamically reconfigurable Fano metamaterial through graphene tuning for switching and sensing applications. Sci. Rep..

[44] Smith D.R., Vier D.C., Koschny T., Soukoulis C.M. (2005). Electromagnetic parameters retrieval from inhomogeneous metamaterials. Phys. Rev. E.

[45] Smith D.R., Schultz S., Markos P., Soukoulis C.M. (2001). Determination of Effective Permittivity and Permeability of Metamaterials from Reflection and Transmission Coefficients. Phys. Rev. B.

[46] Yang M., Liang L., Zhang Z., Xin Y., Yao J. (2019). Electromagnetically induced transparency-like metamaterials for detection of lung cancer cells. Opt. Express.

