# Supplementary material for: Actively Controllable Terahertz Metal–Graphene Metamaterial Based on Electromagnetically Induced Transparency Effect
Source: Nanomaterials (Basel). 2022 Oct 19;12(20):3672. doi: 10.3390/nano12203672 (PMC9609005; doi:10.3390/nano12203672)
Supplement: Supplementary file 1 [file nanomaterials-12-03672-s001.zip › nanomaterials-1953260-supplementary.pdf]

Supplementary Materials: Actively controllable terahertz metal-graphene metamaterial based on electromagnetically induced transparency effect

Liang Gao [1,†], Chao Feng [1,†], Yongfu Li [1], Xiaohan Chen [2,*], Qingpu Wang [1] and Xian Zhao [1,*]

1. Reflected wave and phase properties

The reflection spectra and phase properties of the metal-graphene device with different graphene conductivities, as shown in Figure S1 and Figure S2, respectively, can be obtained simultaneously along with the transmission spectra simulation.

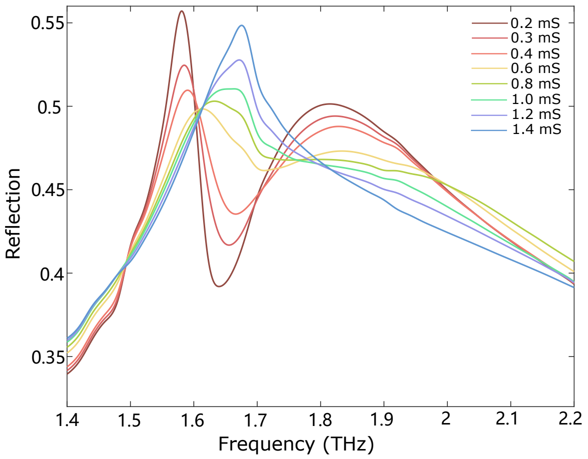

Figure S1. Reflection spectra of the metal-graphene device for different DC graphene conductivities.

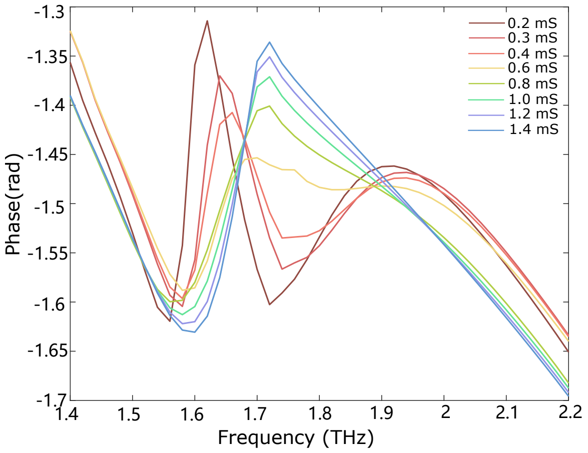

Figure S2. Phase properties of the metal-graphene device for different DC graphene conductivities.

2. Equivalent circuit model

To investigate the resonance condition of the coupled resonators, it is convenient to build an equivalent LCR circuit model [1,2]. The individual resonators can be described as antennas which have a frequency-dependent impedance described. The coupling of the resonators is via a parallel coupling capacitor (C_c), as shown in Figure S3. The capacitor C_1 and C_2 are due to the energy stored in the electric field due to the build up of charges at each end of the resonators. The inductance L_1 and L_2 are due to the magnetic field around the resonator, which stores energy as the current flows. The resonator periodically cycles energy stored between the electric field and the magnetic field, with the resistor values R_1

and R_2 describing the energy lost per cycle from emitting radiation and from ohmic losses in the metal. Extra induced loss by the graphene patch in the dark resonator is described by an added variable resistor in series, R_{Damp}. The voltage sources V_1 and V_2 describes the electromotive force of the incident electric field on the electrons, with the induced current, $I = V/Z$ from this driving voltage determined by considering the overall impedance of the circuit.

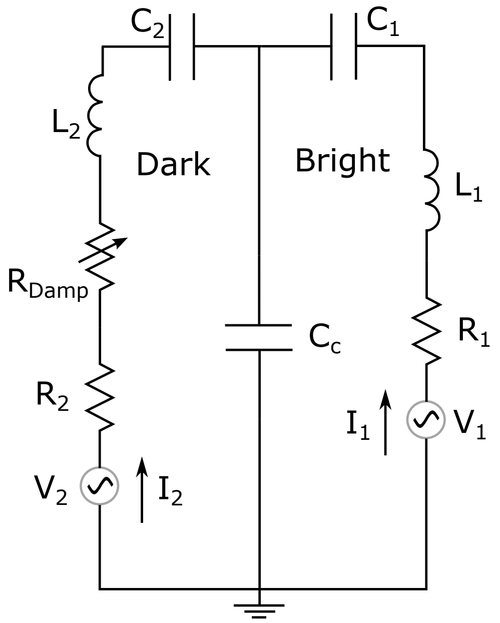

Figure S3. Equivalent circuit model of the metal-graphene coupled devices.

The power drawn, P_{Drawn}, from voltage sources V_1 and V_2, was calculated in order to retrieve the transmission through the metamaterial device as this is proportional to the extinction coefficient and hence is proportional to $|1 - T|$ [3]. To estimate the power drawn, the current from each of the voltage sources as a function of angular frequency is derived as:

$$\begin{pmatrix} i_1 \\ i_2 \end{pmatrix} = (Z)^{-1} \begin{pmatrix} V_1 \\ V_2 \end{pmatrix} \tag{1}$$

$$Z = \begin{pmatrix} R_1 + jL_1\omega + \dfrac{C_1 + C_c}{j\omega C_1 C_c} & \dfrac{1}{j\omega C_c} \\ \dfrac{1}{j\omega C_c} & R_2 + R_{Damp} + jL_2\omega + \dfrac{C_2 + C_c}{j\omega C_2 C_c} \end{pmatrix} \tag{2}$$

The current drawn from the voltage sources can be used to determine the time averaged power drawn as follows

$$P_{Drawn} = \frac{1}{2} Re(V_1 i_1^* + V_2 i_2^*) \tag{3}$$

In this equation, i_1^* and i_2^* describe the complex conjugate of the current induced in the bright and dark resonators. To convert the power drawn to the transmission coefficient of the device, the following equation is used [3]:

$$|S_{21}|^2 = |S_{21}|^2_{sub} - \frac{P_{Drawn}}{P_{Inc}} \tag{4}$$

Where, $|S_{21}|^2_{sub}$ is transmission due to the substrate and is set to 0.75 here. P_{Inc} is the incident power, 1W.

To determine the specific values for the equivalent circuit model, the charges collected in the resonator, q , the voltage, V, between each end of the resonator, the average current distribution in the antenna, I , the magnetic flux, Φ, are probed in the simulation to calculate

the capacitance and inductance values. The resistive loss for the circuit is determined using the Q factor of the circuit. The circuit parameters are list as following:

Circuit Parameters	Value
C_1	2.9×10^{-16} F
C_2	3.2×10^{-16} F
C_c	20×10^{-16} F
R_1	70 Ω
R_2	36 Ω
L_1	2.1×10^{-11}H
L_2	1.4×10^{-11}H

Table S1. The parameter values of the equivalent circuit.

Following the above methods, the calculate transmission spectra based on the equivalent circuit model is shown in Figure S4, which is in agreement with the results in Figure 3a.

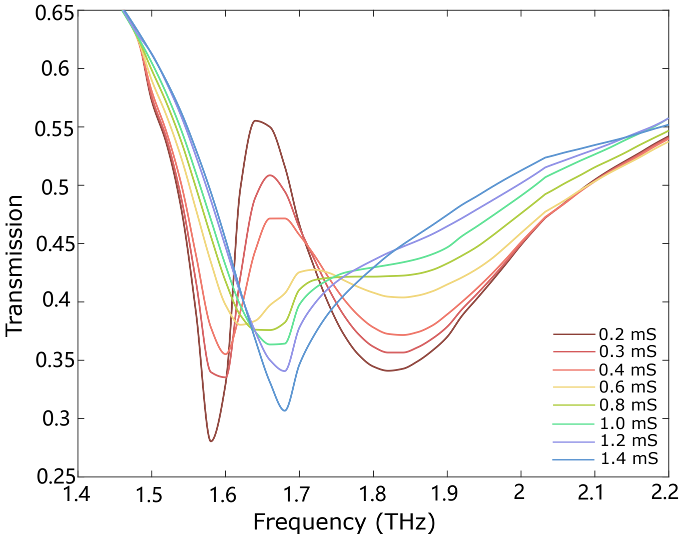

Figure S4. The calculate transmission spectra based on the equivalent circuit model for different DC graphene conductivities.

References

1. Meyrath, T.P.; Zentgraf, T.; Giessen, H. Lorentz model for metamaterials: Optical frequency resonance circuits. *Physical Review B* **2007**, *75*, 205102.
2. García-Vigueras, M.; Mesa, F.; Medina, F.; Rodríguez-Berral, R.; Gómez-Tornero, J.L. Simplified Circuit Model for Arrays of Metallic Dipoles Sandwiched Between Dielectric Slabs Under Arbitrary Incidence. *IEEE Transactions on Antennas and Propagation* **2012**, *60*, 4637–4649.
3. Amin, M.; Farhat, M.; Bagci, H. A dynamically reconfigurable Fano metamaterial through graphene tuning for switching and sensing applications. *Scientific Reports* **2013**, *3*, 2105.
